# Comparison of statistical procedures for estimating polygenic effects using dense genome-wide marker data

**DOI:** 10.1186/1753-6561-3-s1-s12

**Published:** 2009-02-23

**Authors:** Eduardo CG Pimentel, Sven König, Flavio S Schenkel, Henner Simianer

**Affiliations:** 1 Institute of Animal Breeding and Genetics, University of Göttingen, Göttingen, 37075, Germany; 2 Department of Animal and Poultry Science, University of Guelph, Guelph – ON, N1G 2W1, Canada

## Abstract

In this study we compared different statistical procedures for estimating SNP effects using the simulated data set from the XII QTL-MAS workshop. Five procedures were considered and tested in a reference population, i.e., the first four generations, from which phenotypes and genotypes were available. The procedures can be interpreted as variants of ridge regression, with different ways for defining the shrinkage parameter. Comparisons were made with respect to the correlation between genomic and conventional estimated breeding values. Moderate correlations were obtained from all methods. Two of them were used to predict genomic breeding values in the last three generations. Correlations between these and the true breeding values were also moderate. We concluded that the ridge regression procedures applied in this study did not outperform the simple use of a ratio of variances in a mixed model method, both providing moderate accuracies of predicted genomic breeding values.

## Background

The development of appropriate methods to detect a large number of DNA sequence variations in the genome has launched a series of studies [1,2] attempting to associate such alterations with phenotypic variation in complex traits. High-density panels for genotyping thousands of single nucleotide polymorphisms (SNP) are now commercially available and their costs are likely to decrease over time. If the number of markers in such a panel is large enough that it covers the entire genome, then it may be assumed that most of the quantitative trait loci (QTL) associated with a given trait will be in linkage disequilibrium with at least some of these markers. The use of this new source of information in selection programs requires accurate estimation of the effects of QTL associated with the markers, or alternatively the effects of the markers themselves, on traits of interest. Genome-wide estimated breeding values (GEBV) can then be calculated by taking the summation of these effects across the whole genome. Here we compared different statistical approaches to estimate SNP effects using the simulated common data set provided by the XII QTL-MAS workshop.

## Methods

The approach adopted here followed the implementation of genomic selection described in [3]. Selection candidates have their GEBV calculated from a prediction equation. This prediction equation is derived and tested in another sample of animals, not necessarily related to the selection candidates, called the reference population. The reference population comprises a discovery data set, from which the prediction equation is derived; and a validation data set, in which the equation is tested to assess its accuracy. Animals in the reference population must then have both phenotype records and marker genotype information available.

### Data

The available simulated population consisted of 5,865 animals from seven generations. Animals from the first four generations had both phenotypic records and genotypes for the 6,000 SNP loci and therefore were used to form the reference population. The discovery and the validation data sets were defined as the animals belonging to the first three (3,165 animals) and the fourth (1,500 animals) generations, respectively.

Two genetic evaluations were performed: one for the animals in the discovery sample only (GE1); and another for all animals in the reference population (GE2). In both cases, an animal model with a fixed effect of gender was used. Variance components were estimated using VCE [4]. Estimated breeding values (EBV) from GE1 were used as the response variable in the derivation of the prediction equation, in the discovery data set. Then correlations between GEBV and EBV, from GE2, were computed for the animals in the validation data set, and used as reference for comparison among the statistical procedures.

### Model

A multiple linear regression model [2,5] was employed to estimate single additive SNP effects on the estimated genetic merit of animals. The model equation is described below:

$${\text{y}}_{\text{i}}=\mu +\sum_{\text{j}=1}^{\text{p}}{\text{x}}_{\text{ij}}{\text{b}}_{\text{j}}+{\text{e}}_{\text{i}}$$

where:

y_i _is the EBV of the i^th ^animal;

μ is an overall mean;

x_ij _is an indicator variable for the j^th ^SNP genotype of the i^th ^animal;

b_j _is the slope on the j^th ^SNP genotype;

p is the number of genotyped SNPs;

e_i _is a random residual term.

The coefficients x_ij _were defined as -1 for genotype A_1_A_1_, 0 for genotype A_1_A_2 _and +1 for genotype A_2_A_2_. Here we did not make any assumption about the positions of the QTL and assumed strictly additive effects for the markers. Therefore the genomic region represented by a given marker was treated much like a QTL and b_j _was actually an estimate of the QTL allele substitution effect.

### Statistical procedures

If the number of markers is greater than the number of genotyped animals, ordinary or weighted least squares cannot be used to estimate the regression coefficients, unless some variable selection strategy is adopted, which may lead to unsatisfactory results [1]. This lack of degrees of freedom can be overcome if SNP genotype is treated as a random effect and mixed model methodology is employed to obtain best linear unbiased prediction (BLUP) of SNP effects. Another alternative/interpretation is the use of ridge regression (RR) or another form of Bayesian procedure. Consider the following system of equations:

$$\left[\begin{array}{cc} \iota^{t}W\iota & \iota^{t}WX\\ X^{t}W\iota & X^{t}WX+\Phi \end{array}\right]\left[\begin{array}{c} \mu \\ b \end{array}\right]=\left[\begin{array}{c} \iota^{t}Wy\\ X^{t}Wy \end{array}\right]$$

where:

**ι **is a (n × 1) vector of ones, where n is the number of genotyped animals;

**W **is a diagonal matrix with w_ii _equal to the reliability of the EBV of the i^th ^animal;

**X **is the (n × p) matrix of coefficients x_ij_;

**Φ **is a square matrix of order p.

The key point in the estimation process here is the definition of **Φ **and this is the parameter that characterizes the departure from weighted least squares to the following statistical procedures:

BLUP1: **Φ **= **I**

In this method equal variances were assumed for all segments and the ratio of the residual to the segment variances was assumed to be 1, regardless of the heritability of the trait.

$$\begin{array}{ccccc} \text{BLUP}2:\Phi =I\lambda & \text{with} & \lambda =\frac{\sigma_{\text{e}}^{2}}{\sigma_{\text{SNP}}^{2}} & \text{and} & \sigma_{\text{SNP}}^{2}=\frac{\sigma_{\text{a}}^{2}}{\text{p}} \end{array}$$

Here the variances of all segments were also assumed to be equal, but the information on the residual and additive genetic variances, estimated from the discovery sample, was used.

$$\text{RR}1:\begin{array}{ccccc} \Phi =\text{diag}(\begin{array}{cccc} \varphi_{1} & \varphi_{2} & ... & \varphi_{\text{p}} \end{array}) & \text{where} & \varphi_{\text{i}}=\theta \frac{{\text{VIF}}_{\text{i}}}{\max(\text{VIF})} & \text{with} & \text{i}=1,2,...,\text{p} \end{array}$$

VIF stands for Variance Inflation Factor and is defined as: ${\text{VIF}}_{\text{i}}=\frac{1}{1-{\text{r}}_{\text{i}}^{2}}$

where ${\text{r}}_{\text{i}}^{2}$ is the coefficient of determination obtained when the i^th ^covariate is regressed on all other covariates in the model. This is a RR procedure similar to the one implemented in [5]. Here we tested different values of θ, starting from 1.0 with increments of 1.0, and picked the value that yielded the highest correlation between EBV and GEBV, while in [5] a combination of bootstrap with cross validation was used to choose a value of θ that minimized the mean squared error of prediction. Each VIF was calculated as the product of the diagonal element of the left hand side of the equations, by the corresponding diagonal element of its inverse, following [6].

$$\begin{array}{ccccc} \text{RR}2:\Phi =\text{diag}(\begin{array}{cccc} \varphi_{1} & \varphi_{2} & ... & \varphi_{\text{p}} \end{array}) & \text{where} & \varphi_{\text{i}}=\theta \frac{1}{\text{abs}({\text{t}}_{\text{i}})} & \text{with} & \text{i}=1,2,...,\text{p} \end{array}$$

where abs(t_i_) is the absolute Student-t statistic for testing the null hypotheses that the value of the i^th ^parameter is zero. The criterion for choosing the value of θ was the same as above.

RR2*: a variant of RR2 to be done in two steps only (i.e., without testing different values for θ): i) estimate SNP effects with BLUP1; ii) use the t-values of estimates to define the weights. In the second step, ϕ_i _was set either to zero if abs(t_i_) exceeded the mean abs(t_i_) by more than 3 standard deviations, or to λ (used in BLUP2) otherwise.

## Results and discussion

Estimated residual and additive genetic variances were 3.17 and 1.23 within the discovery sample, and 3.12 and 1.36 in whole the reference population, respectively. Within the discovery sample, reliabilities on the EBV ranged from 0.48 to 0.86, with average and standard deviation of 0.50 ± 0.05. The mean (± SD) of the EBV in the validation sample was 0.185 ± 0.844.

**Figure 1:**
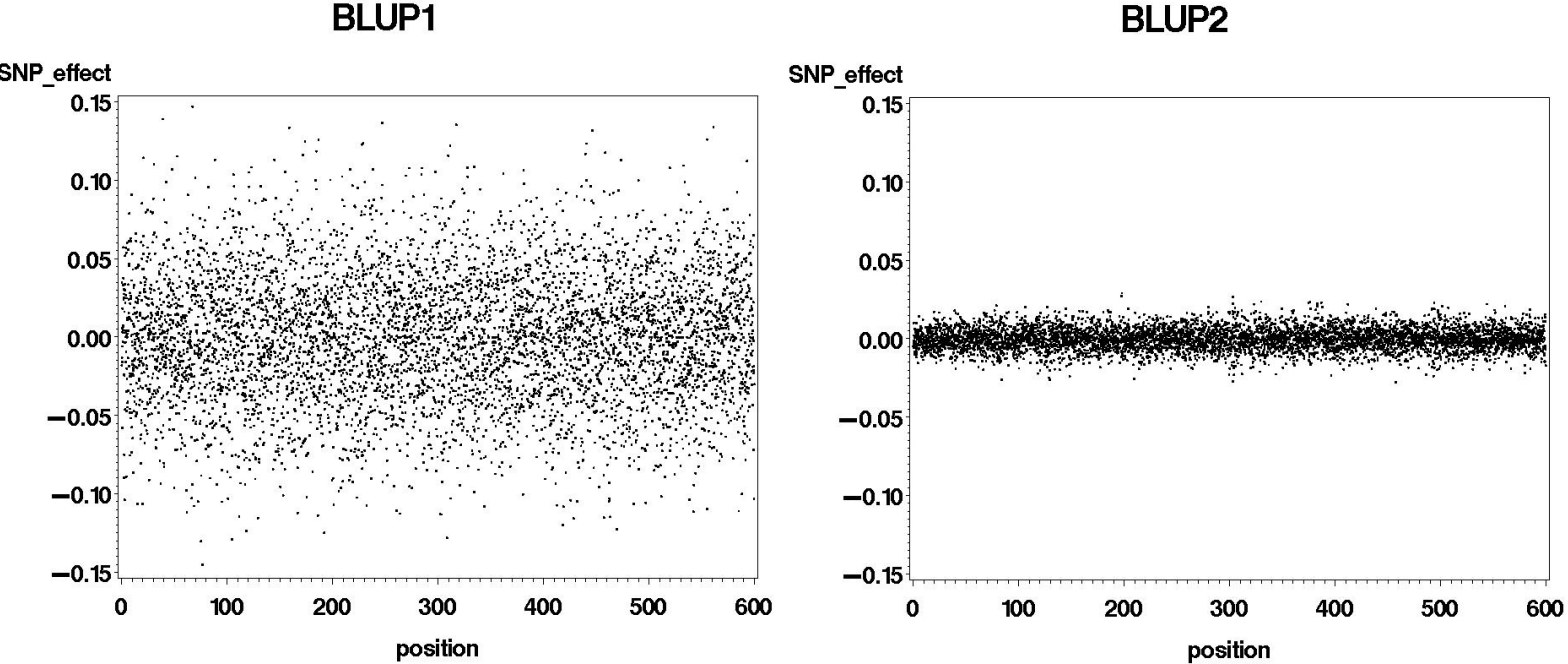
**Marker effects, estimated from alternate BLUP procedures, against position (cM) on the genome**.

**Figure 2:**
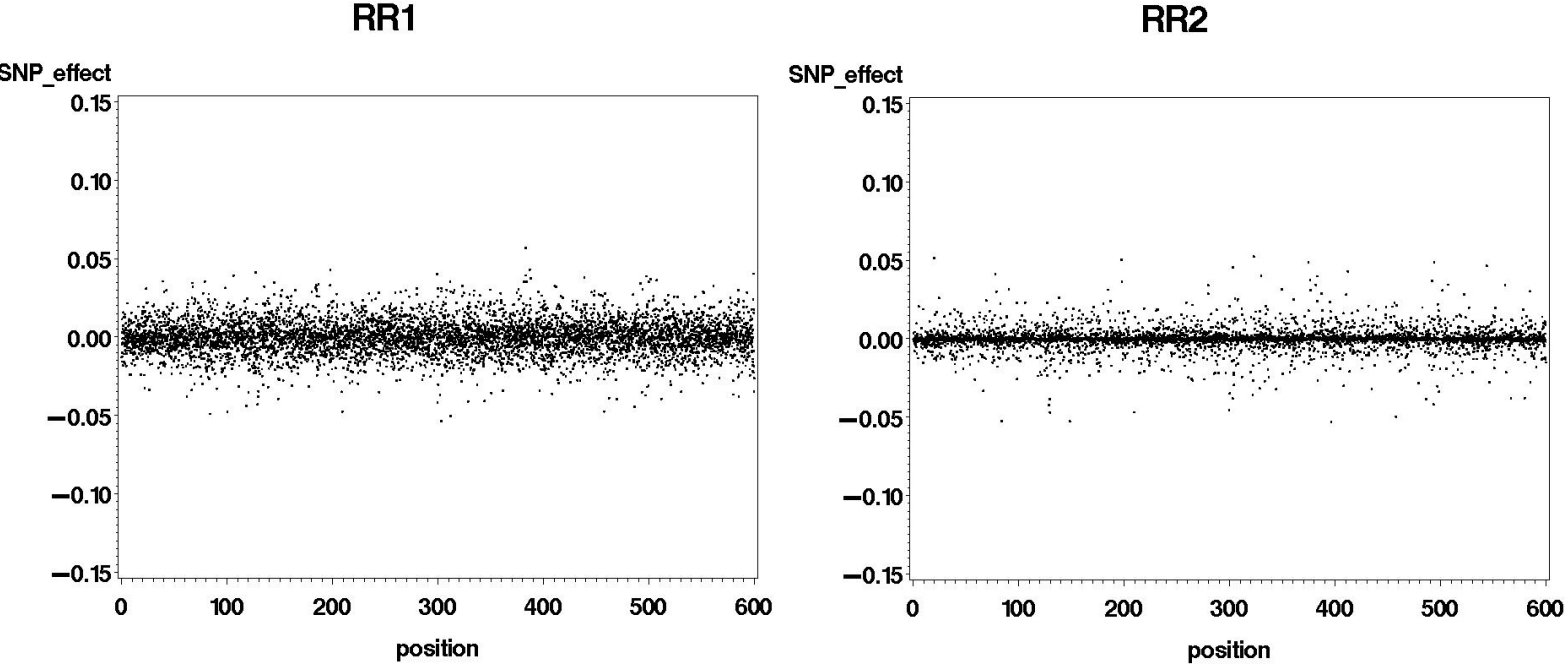
**Marker effects, estimated from alternate ridge regression (RR1 and RR2) procedures, against position (cM) on the genome**.

The means (± SD) of the GEBV and correlations between EBV and GEBV, in the validation sample, from all procedures are presented in Table 1. Estimates of regression coefficients against the marker position on the genome for the first four methods are presented in Figures 1 and 2.

**Table 1:** Means of GEBV and correlations between EBV and GEBV in the validation sample (Generation 3), from each procedure.

	**Mean GEBV ± SD**	**r_EBV, GEBV _± SE**
BLUP1	0.355 ± 0.719	0.499 ± 0.019
BLUP2	0.366 ± 0.550	0.611 ± 0.016
RR1	0.366 ± 0.580	0.588 ± 0.017
RR2	0.360 ± 0.533	0.630 ± 0.016
RR2*	0.363 ± 0.556	0.603 ± 0.016

In both BLUP procedures, equal variances were assumed for all markers. Therefore, the difference between them was due to the amount of shrinkage imposed. In BLUP2, the assumed variance for each marker was very small, which resulted in a large value for the ratio, and a much stronger shrinkage on parameter estimates (Figure 1). The BLUP2 would therefore be closer to a prior assumption that marker effects are expected to be close to zero, not allowing some of them to deviate from this expectation. A more realistic assumption would be that QTL effects follow a Gamma distribution, where many have a small effect and few have a large effect, as suggested in [7] and used in [1,2]. In our study, a prior distribution of variances of markers was not formally defined. Instead, the different weights in the RR1 and RR2 procedures were derived from the data, in the form of VIF and t-values.

When different levels of shrinkage were allowed by the weighting factors in the RR1 and RR2 methods some discrimination among marker effects could be made (Figure 2). This feature was more pronounced in RR2, where weights were functions of t-values. These two ridge regression investigative procedures (i.e., testing different values of θ) were used in an attempt to identify one possible parameter to be used in a simpler and faster way. Since RR2 seemed more promising, the t-values were picked as the parameters to be used in the two-step procedure RR2*.

Methods BLUP2 and RR2* were then used to estimate SNP effects again using data from the whole reference population. Correlations between GEBV and the true breeding values in the last three generations ranged from 0.40 in generation 6 to 0.52 in generation 4 (table 2 in [8]). The lower correlation with the true breeding values can in part be explained by the use of EBV as a proxy for breeding values in the analyses performed here. Notice that the average reliability on the EBV in the discovery sample was only 0.5. In a real application, one would likely use highly accurate EBV to derive the prediction equation.

Methods BLUP2 and RR2* were then used to derive prediction equations, using the phenotypes as response variable. Correlations between true breeding values and GEBV predicted with these equations for the last three generations are presented in Table 2. Correlations were slightly higher than when using EBV.

**Table 2:** Correlations between GEBV and true breeding values, when the response variable on the estimation step was the phenotype.

**Method**	**Generation 4**	**Generation 5**	**Generation 6**	**Generations 4–6**
BLUP2	0.55	0.51	0.48	0.51
RR2*	0.53	0.51	0.47	0.49

Results from other methods presented at the Workshop indicated that the definition of priors in a full-fledged Bayesian framework may provide higher accuracies of genomic breeding values.

## Conclusion

The ridge regression procedures applied in this study did not outperform the simple use of a ratio of variances in a mixed model method, both providing moderate accuracies of predicted genomic breeding values.

## Competing interests

The authors declare that they have no competing interests.

## Authors' contributions

ECGP designed and carried out the statistical analyses of the study. SK, FSS and HS contributed to the interpretation and discussion of results and took part in writing the manuscript. All authors have read and approved the final version.

## References

[B1] Meuwissen THE, Hayes BJ, Goddard ME (2001). Prediction of total genetic value using genome-wide dense marker maps. Genetics.

[B2] Xu S (2003). Estimating polygenic effects using markers of the entire genome. Genetics.

[B3] Goddard ME, Hayes BJ (2007). Genomic selection. J Anim Breed Genet.

[B4] Groeneveld E (1998). VCE User's Manual, Version 425.

[B5] Roso VM, Schenkel FS, Miller SP, Schaeffer LR (2005). Estimation of genetic effects in the presence of multicollinearity in multibreed beef cattle evaluation. J Anim Sci.

[B6] Maindonald JH (1984). Statistical computation.

[B7] Hayes B, Goddard ME (2001). The distribution of the effects of genes affecting quantitative traits in livestock. Genet Sel Evol.

[B8] Lund MS, Sahana G, deKoning D-J, Carlborg Ö (2009). Comparison of analyses of the QTLMAS XII common dataset.  I: Genomic selection. BMC Proceedings.

